# Effectiveness and relevant factors of 2 % rebamipide ophthalmic suspension treatment in dry eye

**DOI:** 10.1186/s12886-015-0040-0

**Published:** 2015-06-06

**Authors:** Kaori Ueda, Wataru Matsumiya, Keiko Otsuka, Yoshifumi Maeda, Takayuki Nagai, Makoto Nakamura

**Affiliations:** Division of Ophthalmology, Department of Surgery, Kobe University Graduate School of Medicine, 7-5-2 Kusunoki-cho, Chuo-ku, Kobe, 650-0017 Japan

**Keywords:** Dry eye, Rebamipide, Relevant factor, Dry eye-related symptom, Fluorescein ocular surface staining score

## Abstract

**Background:**

Rebamipide with mucin secretagogue activity was recently approved for the treatment of dry eye. The efficacy and safety in the treatment of rebamipide were shown in two pivotal clinical trials. It was the aim of this study to evaluate the effect of 2 % rebamipide ophthalmic suspension in patients with dry eye and analyze relevant factors for favorable effects of rebamipide in clinical practice.

**Methods:**

This was a retrospective cohort study of 48 eyes from 24 patients with dry eye treated with 2 % rebamipide ophthalmic suspension. Dry eye-related symptom score, tear film break-up time (TBUT), fluorescein ocular surface staining score (FOS) and the Schirmer test were used to collect the data from patients at baseline, and at 2, 4, 8, and 12 week visits. To determine the relevant factors, multiple regression analyses were then performed.

**Results:**

Mean dry eye-related symptom score showed a significant improvement from the baseline (14.5 points) at 2, 4, 8 and 12 weeks (9.80, 7.04, 7.04 and 7.83 points, corrected P value <0.001, respectively). Median FOS showed a significant improvement from the baseline (3.0 points) at 2, 4, 8 and 12 weeks (2.0, 2.0, 1.0 and 1.0 points, corrected P value <0.001, respectively). TBUT and Schirmer test values were not significantly improved after the treatment. For ocular symptoms, three parameters (foreign body sensation, dry eye sensation and ocular discomfort) showed significant improvements at all visits. The multiple regression analyses showed that the fluorescein conjunctiva staining score was significantly correlated with the changes of dry eye-related symptom score at 12 weeks (P value = 0.017) and dry eye-related symptom score was significantly correlated with independent variables for the changes of FOS at 12 weeks (P value = 0.0097).

**Conclusions:**

Two percent rebamipide ophthalmic suspension was an effective therapy for dry eye patients. Moreover the fluorescein conjunctiva staining score and dry eye-related symptom score might be good relevant factors for favorable effects of rebamipide.

**Electronic supplementary material:**

The online version of this article (doi:10.1186/s12886-015-0040-0) contains supplementary material, which is available to authorized users.

## Background

Dry eye is an important public health problem causing ocular discomfort, fatigue, and visual disturbance that may interfere with daily activities [1]. Current thinking is expressed in the definition presented by the Definition and Classification Subcommittee of the International Dry Eye WorkShop Report [2]: Dry eye is “a multifactorial disease of the tears and ocular surface that results in symptoms of discomfort, visual disturbance, and tear film instability with potential damage to the ocular surface. It is accompanied by increased osmolarity of the tear film and inflammation of the ocular surface”. [2]. Based on data from the largest epidemiological studies of dry eye, the Women’s Health Study, and other studies, it has been estimated that about 7.8 % or 3.23 million American women and 4.7 % or 1.6 million men >50 years old have dry eye disease [3, 4]. Dry eye is also one of the most prevalent eye diseases especially in Asia, where as many as 20–50 % of the population of older people in some areas may be affected [5]. In Japan, Uchino et al. showed that the prevalence for the combination of clinically diagnosed dry eye and severe symptoms of dry eye in men and women were 12.5 % and 21.6 %, respectively. These were almost two-fold higher than the prevalence reported by Schaumberg using the same questionnaires and diagnostic criteria in the United States [3, 6]. Therefore, dry eye is one of the most common ophthalmological problems in Japan also. The tear film has been traditionally reported to consist of three important components: a mucin layer that coats the ocular surface epithelium, an aqueous layer that is present between the mucin and a lipid layer, and a lipid layer that overlays the surface of the tear film [7]. The destabilization of the tear film caused by decreased tear production or altered tear composition can induce ocular surface damage, inflammation and ultimately further tear film instability. Therefore, the lack of mucins can reduce the stability of the tear film and lead to or aggravate dry eye disease [8]. The main objectives in caring for patients with dry eye disease are to improve their ocular comfort and quality of life, and to return the ocular surface and its film to the normal homeostatic state. Recently, the safety and efficacy of diquafosol eye drops to stimulate both water and mucin secretion as an agonist of the purinergic P2Y2 receptor has been favorably evaluated in several experimental and clinical trials. On the other hand, rebamipide is also a quinolinone derivative with mucin secretagogue activity and rebamipide ophthalmic suspension (Mucosta ophthalmic suspension UD2 %; Otsuka Pharmaceutical Co. Tokyo, Japan) was recently approved for the treatment of dry eye in Japan. The therapeutic effects of rebamipide ophthalmic suspension are considered to be due to the increase of corneal and conjunctival mucin, and its safety and efficacy have been established in clinical trials and some experimental reports. In this observational case series study, we have evaluated the effect of 2 % rebamipide ophthalmic suspension in patients with dry eye at baseline, and at 2, 4, 8, and 12 week visits in order to elucidate the therapeutic effects of rebamipide ophthalmic suspension in clinical practice and shown relevant factors for the changes of subjective signs and objective symptoms of dry eye.

## Methods

This was a retrospective case series study. All participants in this study were Japanese individuals recruited from the Department of Ophthalmology at the Kobe University Hospital in Japan. The study was approved by the Institutional Review Board of Kobe University Graduate School of Medicine and adhered to the tenets that were set forth in the Declaration of Helsinki. The diagnosis of dry eye was based on the diagnostic criteria of the Dry Eye Research group in Japan [9]. Briefly, patients with three essential problems, which were dry eye-related symptoms, abnormality of tear stability or secretion, and ocular surface damage were regarded as suffering from dry eye. Patients with two out of three problems were regarded as suspected of suffering from dry eye. The tear secretion was regarded as abnormal if the Schirmer I test resulted in equal to or less than 5 mm, and the tear stability was regarded as abnormal if TBUT (tear break up time) values resulted in equal to or less than 5 mm. The ocular surface damage was regarded as existing if the van Bijsterveld system score was equal to more than three points. In the current study, patients with two or three problems were enrolled. The ocular surface and anterior segment examinations with a slit lamp were performed at every visit. In addition, fluorescein ocular surface staining (FOS), TBUT measurement, Schirmer’s test and interviews related to subjective dry eye symptoms were also performed as far as possible at every visit. Clinical data were assessed at the following times: baseline visit and visits at 2 weeks, 4 weeks, 8 weeks, and 12 weeks. Patients with cicatricial keratoconjunctival diseases, blepharitis, ocular allergy, meibomian gland disease, corneal hypoesthesia or those who had undergone penetrating keratoplasty or were wearing contact lenses were excluded from this study.

### Patients’ characteristics

Forty-eight eyes from 24 patients with dry eye were recruited between January 2012 and June 2012. All patients were prescribed 2 % rebamipide ophthalmic suspension to be administered four times in a day and followed up for at least 12 weeks. 13 patients were not fully relieved by conventional treatments and received rebamipide ophthalmic suspension as an additional treatment.

### Assessment

The primary endpoints for efficacy were FOS assessment and subjective dry eye related symptom score assessment on each occasion. Moreover, relevant factors for the changes of dry eye related symptom scores and fluorescein ocular surface staining score from baseline to 12 weeks were assessed.

In the present study, fluorescein was instilled by means of a fluorescein　strip (FLUORES Ocular Examination Test Paper 0.7 mg, Showa Yakuhin Kako Co. LTD., Tokyo, Japan) wetted with a drop of saline. Then the strip was tightly shaken once after saline moistening to remove excess fluorescein solution from the strip. The examiner gently retracted the patient’s lower lid and touched the edge of that with the strip. [10] Fluorescein staining was evaluated with a blue free barrier filter according to the van Bijsterveld system, which divided the ocular surface into three zones: nasal bulbar conjunctiva, temporal bulbar conjunctiva, and cornea [11]. Each zone was evaluated on a scale of 0 to 3, with 0 indicating no staining, 1 indicating few separated spots, 2 indicating many separated spots and 3 indicating confluent staining; the maximum possible score with this system was 9. The subjective dry eye symptom was evaluated by conducting interviews covering same 11 parameters, which included foreign body sensation (as a feeling that something is in the eye), photophobia, itching, eye pain, dryness (as a dry sensation in the eye), heaviness, blurred vision, eye fatigue, eye discomfort (as a vaguely felling unpleasant sensation), eye discharge and lacrimation, in the same manner as the parameters were used in the previous reports [12, 13]. Each of the parameters was scored on a 4-point scale from 0 to 3 as follows: 0 = no symptoms, 1 = mild, 2 = moderate, and 3 = severe. Secondary endpoints were TBUT, Schirmer’s test measurement (without topical anesthesia), fluorescein staining score in cornea or conjunctiva, and each parameter score of subjective dry eye symptom on each occasion. We measured tear break-up time (TBUT) using the conventional strip method to assess tear film stability. The time from normal blinking to the first appearance of a dry spot in the tear film was measured three times. The Schirmer’s test was performed without anesthesia to measure tear volume.

Moreover, as possible relevant factors for the changes of dry eye related symptom scores and fluorescein ocular surface staining score from baseline to 12 weeks, first we examined gender, age, fluorescein ocular surface staining score, fluorescein conjunctiva staining score, fluorescein corneal staining score, TBUT, Schirmer’s test, dry eye related symptom scores, the presence of previous treatment, the presence of Sjogren syndrome and the presence of auto-immune disease at baseline using simple regression analyses.

### Statistical analysis

The parametric or nonparametric tests were selected in all analyses with descriptive statistics and statistical testing according to the results of the Shapiro-Wilk test. Values were appropriately described as mean ± SD or median (IQR) according to whether the data were normal distribution, or not. The statistical analyses were performed using MedCalc v.15.2.2 software (MedCalc Software, Mariakerke, Belgium). To determine the factors useful for predicting the changes of objective signs and subjective symptoms related to dry eye, multiple regression analyses using the stepwise method were then performed using the variables that showed some trend toward significant association (P value < 0.2) in the simple regression analyses. At the same time, analyzing correlation coefficient (r) in the all parameters was used by Pearson’s Product–moment Correlation analyses. In the regression analysis, any scores were selected in one eye with worse fluorescein ocular surface staining scores or in the right eye if both eyes scored the same. Missing data of only Schirmer’s test values were dealt with mean imputation (which was the replacement of a missing observation with the mean of the non-missing observations) [14]. One sample paired *t*-test or Wilcoxon’s signed rank test was primarily performed the time course analysis with comparing any two groups (the baseline and each visit time). Though P values of 0.05 or less were considered to be statistically significant, P values were corrected using the Bonferroni method as appropriate. Moreover, Repeated measures analysis of variances or Friedman test were appropriately used to analyze overall trend in the changes of the parameter throughout the present study.

## Results

**Fig. 1:**
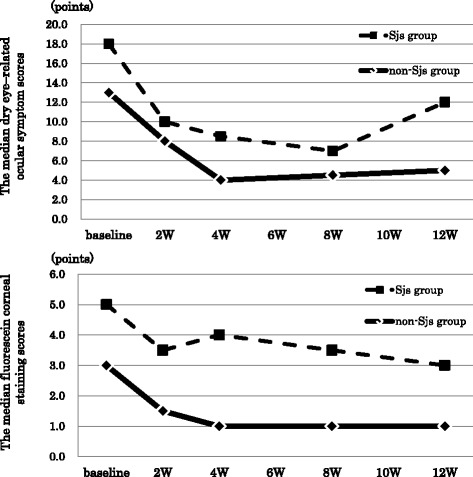
The scores of efficacy endpoints in Sjogren syndrome and non- Sjogren syndrome. The top row shows the time course analyses of dry eye–related ocular symptom scores at baseline, and at 2, 4, 8, and 12 week visits in Sjogren syndrome and non- Sjogren syndrome. The bottom row shows the time course analyses of fluorescein corneal staining scores at baseline, and at 2, 4, 8, and 12 week visits in Sjogren syndrome and non- Sjogren syndrome. Squares with dashed lines: Sjogren syndrome group. Diamonds with solid lines: non- Sjogren syndrome group; Values represent median. Corrected P value using the Bonferroni method was represented by Wilcoxon’s signed rank test. **P’ < 0.01 ***P’ < 0.001, P’ = corrected P value

The data summary of baseline characteristics is shown in Table 1 and the data for each patient are shown in the Additional file 1: Table S1. Thirteen patients (54.2 %) had received previous treatment for dry eye, and all of them used 2 % rebamipide ophthalmic suspension as an additional treatment in this study. In seven patients (29.1 %) with Sjogren syndrome, three patients were primary Sjogren syndrome, and four patients were secondary Sjogren syndrome: three had rheumatoid arthritis and one had systemic lupus erythematosus. The time course analysis of efficacy endpoints is shown in Table 2. In the analyses of repeated measures, all parameters except for TBUT were also significantly improved. Besides, Mean dry eye symptom score and median FOS showed a significant improvement from the baseline at 2, 4, 8 and 12 weeks (corrected P value <0.001 at each time point). Moreover, fluorescein corneal staining score and fluorescein conjunctiva staining score were significantly improved at each time point. However TBUT was significantly improved only at 8 weeks and Schirmer’s test values were not significantly improved after the treatment. The time course analysis of 11 parameters of dry eye related symptom is shown in Table 3. The parameters except for itching, eye discharge and tearing were significantly improved in the analyses of repeated measures. In particular, three parameters (foreign body sensation, dry eye sensation and ocular discomfort) showed significant improvements at all visits as for ocular symptoms. Eye pain showed significant improvements at 2, 4 and 8 weeks, and approaching significant improvement at 12 weeks (corrected P value =0.064). In the non-Sjogren syndrome (non-SS) group, median dry eye symptom score and FOS showed significant improvements at all visits. They were also significantly improved in the analyses of repeated measures. On the other hand, in the Sjogren syndrome (SS) group, median dry eye symptom score and fluorescein ocular surface staining score did not show significant improvements, though those showed improvements from baseline at all visits (Fig. 1) and were significantly improved in the analyses of repeated measures. Multiple regression analyses of independent variables for the changes of dry eye related symptom scores and fluorescein ocular surface staining score from baseline to 12 weeks are shown in Table 4. It was shown that fluorescein conjunctiva staining score was significantly correlated with the changes of dry eye related symptom scores from baseline to 12 weeks (*r* = −0.48, P value = 0.017). Moreover, FOS, Schirmer’s test, dry eye symptom score and the presence of previous treatment had mild correlation with those (*r* = −0.45, *r* = 0.39, *r* = −0.47, *r* = −0.45, respectively), though they were not included in the stepwise model. On the other hand, it was observed that dry eye symptom score was significantly correlated with the changes of fluorescein ocular surface staining score from baseline to 12 weeks (*r* = −0.52, P value = 0.0097). Moreover, FOS, fluorescein conjunctiva staining score and the presence of previous treatment were mildly correlated with those (*r* = −0.38, *r* = −0.37, *r* = −0.32, respectively), though they were not included in the stepwise model. The most frequently observed adverse event was bitter taste in this study. This was observed in 17 patients (70.8 %) out of the total of 24. Itching and stinging sensation were observed in one patient and two patients, respectively.

**Table 1:** Baseline characteristics

Sex	Female	21 (87.5 %)
Age (yrs)	Mean	66.2
Dry eye-related symptom score	Mean	14.5
Fluorescein ocular surface staining score	Median R/L	3.0 / 3.0
TBUT (sec)	Median R/L	2.0 / 2.0
Schirmer’s test (mm)	Median R/L	6.0 / 6.0
Previous treatment for dry eye		n (%)
	none	11 (45.8 %)
	AT	3 (12.5 %)
	SH	8 (33.3 %)
	SH + AT	2 (8.3 %)
Primary disease of dry eye		n (%)
non-Sjogren group n = 17	RA	1 (5.8 %)
	Basedow disease	1 (5.8 %)
	any other autoimmune disease	4 (23.5 %)
	none	11 (64.7 %)
Sjogren group n = 7	Prymary	3 (42.8 %)
	Secondary	4 (57.1 %)

TBUT; tear break-up time, AT; artificial tears, SH; sodium hyaluronate, RA; rheumatoid arthritis, Vales are shown as mean where applicable

**Table 2:** The scores of efficacy endpoints at baseline, and at 2, 4, 8, and 12 week visits

Total		Baseline	At 2 week	At 4 week	At 8 week	At 12 week
Dry eye symptom score		14.5 ± 6.97	9.80 ± 5.26 ***	7.04 ± 5.42 ***	7.04 ± 6.46 ***	7.83 ± 6.82 ***
Fluorescein ocular surface staining score		3.0 (2.0–5.3)	2.0 (1.0–3.0)***	2.0 (1.0–4.0)***	1.0 (0.0–3.0)***	1.0 (0.0–3.0)***
	Fluorescein cornea staining score	1.0 (1.0–2.0)	1.0 (0.0–1.0)**	1.0 (0.3–1.0)*	1.0 (0.0–1.0)**	1.0 (0.0–1.0)***
	Fluorescein conjunctiva staining score	2.0 (1.0–3.0)	1.0 (0.0–2.0)**	1.0 (0.0–2.8)**	0.0 (0.0–2.0)**	1.0 (0.0–2.0)**
TBUT		2.5 (2.0–3.0)	3.0 (2.0–3.0)	3.0 (2.0–3.0)	3.0 (3.0–4.0)*	3.0 (2.0–3.3)
Schirmer’s test		6.0 (3.0–10.3)	7.5 (3.5–12.4)	9.0 (4.8–13.0)	7.0 (4.0–12.3)	6.5 (4.0–10.3)

Values are mean ± SD or median (IQR)

*P’ < 0.05, **P’ < 0.01, ***P’ < 0.001

P’ = corrected P value with Bonferroni method

† one sample paired t-test were performed in the analyses of only dry eye symptom score, and Wilcoxon signed-rank tests were performed in the analyses of any other items

**Table 3:** The median scores of 11 parameters about dry eye related symptom at baseline, and at 2, 4, 8, and 12 week visits

Ocular symptom	Baseline	At 2 week	At 4 week	At 8 week	At 12 week
Foreign body sensation	1.5 (0.8–2.3)	1.0 (0.0–1.3)*	0.0 (0.0–1.0)**	0.0 (0.0–1.0)**	0.0 (0.0–1.0)**
Photophobia	2.0 (0.8–2.3)	1.5 (0.8–2.0)	1.0 (0.0–2.0)**	0.0 (0.0–1.0)**	0.5 (0.0–1.3)
Itching	0.0 (0.0–1.0)	0.0 (0.0–0.0)	0.0 (0.0–1.0)	0.0 (0.0–1.0)	0.0 (0.0–0.3)
Eye pain	1.0 (0.0–3.0)	1.0 (0.0–1.0)*	0.0 (0.0–1.0)*	0.0 (0.0–1.0)*	0.0 (0.0–1.0)
Dry eye sensation	2.5 (1.0–3.0)	1.0 (0.8–2.0)*	1.0 (0.0–1.5)**	1.0 (0.0–2.0)**	1.0 (0.0–2.3)**
Heaviness	1.0 (0.0–2.0)	0.0 (0.0–1.0)*	0.0 (0.0–0.5)*	0.0 (0.0–0.0)	0.5 (0.0–1.0)
Blurred vision	2.0 (0.0–2.0)	1.0 (0.0–2.0)	1.0 (0.0–2.0)	0.0 (0.0–2.0)	0.5 (0.0–2.0)
Asthenopia	2.0 (1.0–2.0)	1.0 (1.0–2.0)	1.0 (0.0–2.0)	1.0 (0.0–2.0)*	1.0 (0.0–2.0)*
Ocular discomfort	2.0 (0.8–3.0)	1.0 (0.0–2.0)*	1.0 (0.0–1.0)**	1.0 (0.0–1.5)**	0.0 (0.0–1.0)**
Eye discharge	0.5 (0.3–1.5)	0.0 (0.0–1.0)	0.0 (0.0–1.0)	0.0 (0.0–1.0)	0.0 (0.0–1.0)
Tearing	0.5 (0.0–0.3)	0.0 (0.0–0.0)	0.0 (0.0–0.5)	0.0 (0.0–0.0)	0.0 (0.0–0.0)

Values are median (IQR)

* P’ < 0.05, ** P’ < 0.01

P’ = corrected P value with Bonferroni method

**Table 4:** Independent variables for the changes of dry eye related symptom scores and fluorescein ocular surface staining score from baseline to 12 weeks

a) Independent variables for the changes of dry eye related symptom scores						
	The changes of dry eye related symptom scores from baseline to 12 weeks					
	Crude			Multivariate		
	Unstandardized regression coefficient	Correlation coefficient(r)	P value	Unstandardized regression coefficient	Correlation coefficient(r)	P value
Age	−0.06	−0.092	0.67			
Gender (male)	4.71	0.24	0.25			
Sjogren syndrome	−1.37	−0.097	0.65			
The presence of previous treatment	−5.71	−0.45	0.029			
Dry eye symptom score	−0.44	−0.47	0.021			
Schirmer’s test	0.048	0.39	0.30			
Fluorescein ocular surface staining score	−1.26	−0.45	0.028			
Fluorescein corneal staining score	−1.81	−0.12	0.25			
Fluorescein conjunctiva staining score	−1.81	−0.48	0.017	−1.81	−0.48	0.017*
TBUT	−0.7955	−0.1192	0.5791			
The presence of auto-immune disease	−0.589	−0.045	0.83			
b) Independent variables for the changes of fluorescein ocular surface staining score						
	The changes of fluorescein ocular surface staining score from baseline to 12 weeks					
	Crude			Multivariate		
	Unstandardized regression coefficient	Correlation coefficient(r)	P value	Unstandardized regression coefficient	Correlation coefficient(r)	P value
Age	−0.019	−0.14	0.51			
gender (male)	−0.38	−0.095	0.66			
Sjogren syndrome	−0.20	−0.069	0.75			
The presence of previous treatment	−0.84	−0.32	0.13			
Dry eye symptom score	−0.10	−0.52	0.0097	−0.10	−0.52	0.0097*
Schirmer’s test	0.048	0.22	0.32			
Fluorescein ocular surface staining score	−0.22	−0.38	0.070			
Fluorescein corneal staining score	−0.39	−0.25	0.23			
Fluorescein conjunctiva staining score	−0.29	−0.37	0.076			
TBUT	−0.036	−0.029	0.89			
The presence of auto-immune disease	0.17	0.064	0.77			

*The significant independent variable in the multiple regression analysis

## Discussion

In the current study, dry eye symptom score and fluorescein ocular surface staining score were significantly improved at 2 weeks, 4 weeks, 8 weeks and 12 weeks as a result of treatment with 2 % rebamipide ophthalmic suspension. Previous pivotal clinical trials and other experimental studies have demonstrated the effectiveness of 2 % rebamipide ophthalmic suspension over both short and long term periods [15–18]. However it had not yet been shown which factors correlated with favorable effects of rebamipide for dry eye. The current study showed the effectiveness of 2 % rebamipide ophthalmic suspension for improving the dry eye and furthermore the relevant factors for favorable effects of rebamipide. In fact, it showed that fluorescein conjunctiva staining score at baseline was significantly correlated with the changes of dry eye related symptom scores from baseline to 12 weeks and dry eye symptom score at baseline was significantly correlated with the changes of fluorescein ocular surface staining score from baseline to 12 weeks.

In dry eye patients, the level of membrane-associated mucins, MUC16, in conjunctival epithelial cells is altered [8]. Besides, the level of MUC5AC as secreted mucins in tear fluid has been found to be reduced in individuals with Sjogren’s syndrome [19]. Therefore, 2 % rebamipide ophthalmic suspension with secretion of mucins could be an effective treatment for dry eye by making up for the loss of mucins. In the current study, rebamipide ophthalmic suspension improved objective and subjective ocular surface problems in dry eye patients. Moreover, the effect of rebamipide occurred at 2 weeks after the first treatment as quickly as in previous reports [15–17], and was maintained throughout the 12 weeks.

In dry eye related symptoms, three parameters (foreign body sensation, dry eye sensation and ocular discomfort) showed significant improvements at all visits. In addition, eye pain showed approaching significant improvement at all visits. These results were similar to previous trials [15–17]. These improvements in subjective symptoms should contribute to improved quality of life in patients with dry eye.

Moreover, independent variables for the changes of dry eye related symptom scores and fluorescein ocular surface staining score from baseline to 12 weeks were analyzed with the regression analyses. Consequently, the worse the fluorescein conjunctiva staining score at baseline was, the better the changes of dry eye related symptom scores were. The conjunctival goblet cell density reflected the severity of local disease in the mucin deficient dry eye syndromes [20]. Rebamipide was expected to have favorable effects for conjunctival disorder in short supply of mucins, so that it had secretory ability of mucins and increased conjunctival goblet cell [21, 22]. Therefore rebamipide might make dry eye related symptom relieved. On the one hand, it was reported that the conjunctival inflammation and reduced goblet cell density of dry eye was exacerbated by use of preserved topical agents [20]. Favorable effects of rebamipide for the conjunctival disorder in dry eye patients might be due to not only mucin secreted agent, but also non-preserved topical agent.

On the other hand, the worse dry eye related symptom scores at baseline were, the better the changes of fluorescein ocular surface staining score were in the current study. It was reported that dry eye disease was associated with decreased levels of mucin and an overexpression of IL-6, which correlated with the symptomatic severity of disease [23]. Rebamipide with mucin secretagogue agent was showed to suppress ocular surface inflammation by suppressing the production of cytokines by ocular surface epithelial cells [24]. Thus, the administration of rebamipide for severe dry eye related symptom patients might make fluorescein ocular surface staining score better so that rebamipide would provide mucins and regulate inflammation in the dry eye. The current study did not show that the changes of dry eye related symptom scores and fluorescein ocular surface staining score were associated with whether patients had Sjogren syndrome or not. However Fujita et al. suggested the possibility of a different cause of dry eye between SS dry eye patients and non-SS dry eye patients in the study of dry eye in rheumatoid arthritis patients [25]. Lee SY et al. also suggested that differences in tear cytokine levels as IL-17, TNF-α and IL-6 between SS dry eye and non-SS dry eye patients demonstrated the involvement of different inflammatory processes as causes of dry eye syndrome [26].

Presently, it was not elucidated whether or not the efficacy of 2 % rebamipide ophthalmic suspension was different between non-SS and SS. The presence of previous treatment showed the possibility of a correlation with the improvement of dry eye related symptom scores and fluorescein ocular surface staining score, though it was excluded in the multiple regression model. Therefore the combination therapy with rebamipide and other treatment might have a synergistic effect. However we require a large number size study to resolve these matters, because the current study had some limitation in statistical power. In the current study, 17 patients (70.8 %) out of the total of 24 claimed bitter taste. This was a higher percentage than the 9.7 % in the phase III trial and the 15.7 % in the phase II trial. It was not apparent why the discrepancy in the frequency of bitter taste between the current study and the Phase II and III trials existed. However, it was considered reasonable and proper that the bitter taste was the most frequent adverse effect, because that was associated with the active ingredient [15]. The limitations of the current study are the small sample size, the retrospective design and the lack of a control group. Further studies will be required to investigate which factors correlate with the favorable effects of rebamipide for dry eye.

## Conclusion

In conclusion, 2 % rebamipide ophthalmic suspension is an effective treatment for dry eye. Moreover, worse dry eye related symptom scores might be related to the improvement of objective ocular surface damage and a worse fluorescein conjunctiva staining score might be related to the improvement of subjective dry eye related symptom.

## Additional file

Additional file 1: Table S1. Baseline characteristics in each patient.
