# Supplementary material for: Effectiveness and relevant factors of 2 % rebamipide ophthalmic suspension treatment in dry eye
Source: BMC Ophthalmol. 2015 Jun 6;15:58. doi: 10.1186/s12886-015-0040-0 (PMC4456696; doi:10.1186/s12886-015-0040-0)
Supplement: Additional file 1: Table S1. — Baseline characteristics in each patient. [file 12886_2015_40_MOESM1_ESM.doc]

Additional file 1: Table S1. Baseline characteristics in each patient.

| No | non-SS/SS | Pretreat for Dry eye | Sex | Age | Autoimmune disease | TBUT R/L | Schirmer R/L | symptom scores | FOS R/L |
| --- | --- | --- | --- | --- | --- | --- | --- | --- | --- |
| 1 | non-SS | none | F | 61 | none | 4/4 | NA | 8 | 4/4 |
| 2 | non-SS | none | M | 78 | none | 1/5 | 6/14 | 6 | 2/2 |
| 3 | non-SS | SH+AT | F | 62 | RA | 2/2 | 2/2 | 5 | 6/5 |
| 4 | non-SS | SH | F | 82 | VKH | 4/3 | 2.5/2.5 | 22 | 4/3 |
| 5 | non-SS | SH | F | 62 | none | 3/1 | 3/3 | 23 | 6/6 |
| 6 | non-SS | none | F | 84 | none | 4/3 | 8/6 | 3 | 2/1 |
| 7 | non-SS | none | F | 50 | BD | 1/1 | 30/14 | 12 | 3/3 |
| 8 | non-SS | none | F | 73 | HT | 2/3 | 7/7 | 15 | 1/1 |
| 9 | non-SS | SH+AT | F | 66 | none | 2/2 | 5/0 | 26 | 8/8 |
| 10 | non-SS | none | F | 74 | none | 2/3 | 13/11 | 5 | 1/2 |
| 11 | non-SS | none | M | 65 | none | 2/2 | 10/18 | 15 | 0/0 |
| 12 | non-SS | SH | F | 78 | TA | 2/2 | 5/13 | 15 | 1/3 |
| 13 | non-SS | none | F | 62 | none | 4/4 | 7/3 | 21 | 2/3 |
| 14 | non-SS | SH | F | 65 | PV | 2/1 | 23/13 | 6 | 2/4 |
| 15 | non-SS | none | F | 51 | none | 2/2 | NA | 12 | 5/4 |
| 16 | non-SS | none | F | 70 | none | 3/3 | 11/6 | 19 | 3/2 |
| 17 | non-SS | none | F | 70 | none | 4/3 | 6/6 | 13 | 1/2 |
| 18 | SS | AT | F | 40 | SLE | 2/3 | NA | 14 | 6/6 |
| 19 | SS | AT | F | 63 | none | 3/3 | 7/4 | 13 | 4/3 |
| 20 | SS | SH | F | 61 | RA | 1/1 | NA | 8 | 2/2 |
| 21 | SS | AT | F | 66 | RA | 3/3 | 3/7 | 25 | 7/8 |
| 22 | SS | SH | M | 73 | none | 2/4 | 2/7 | 22 | 8/3 |
| 23 | SS | SH | F | 66 | none | 2/2 | 3/3 | 18 | 7/6 |
| 24 | SS | SH | F | 66 | RA | 3/5 | 3/3 | 22 | 1/2 |

SS; Sjogren syndrome, TBUT; tear break-up time, FOS; fluorescein ocular surface staining score, AT; artificial tears, SH; sodium hyaluronate, RA; rheumatoido arthritis, BD:Basedow disease, HT:Hashimoto's thyroiditis, VKH; Vogt-Koyanagi- Harada disease, SLE; systemic lupus erythematosus, TA; Takayasu's arteritis, PV; Pemphigus vulgaris
